# A Hybrid Data-Driven Metaheuristic Framework to Optimize Strain of Lattice Structures Proceeded by Additive Manufacturing

**DOI:** 10.3390/mi14101924

**Published:** 2023-10-13

**Authors:** Tao Zhang, Uzair Sajjad, Akash Sengupta, Mubasher Ali, Muhammad Sultan, Khalid Hamid

**Affiliations:** 1 School of 3D Printing, Xinxiang University, Xinxiang 453003, China; 2 Department of Energy and Refrigerating Air-Conditioning Engineering, National Taipei University of Technology, Taipei 10608, Taiwan; energyengineer01@gmail.com; 3 Department of Mechanical Engineering, National Yang Ming Chiao Tung University, 1001 University Road, Hsinchu 300, Taiwan; a.sendad@gmail.com; 4 Department of Mechanical and Automation Engineering, The Chinese University of Hong Kong, Hong Kong, China; mubashersuit.edu.pk@gmail.com; 5 Department of Agricultural Engineering, Bahauddin Zakariya University, Bosan Road, Multan 60800, Pakistan; muhammadsultan@bzu.edu.pk; 6 Department of Energy and Process Engineering, Norwegian University of Science and Technology (NTNU), 7491 Trondheim, Norway

**Keywords:** genetic algorithm, deep learning, additive manufacturing, lattice structure, topology optimization

## Abstract

This research is centered on optimizing the mechanical properties of additively manufactured (AM) lattice structures via strain optimization by controlling different design and process parameters such as stress, unit cell size, total height, width, and relative density. In this regard, numerous topologies, including sea urchin (open cell) structure, honeycomb, and Kelvin structures simple, round, and crossbar (2 × 2), were considered that were fabricated using different materials such as plastics (PLA, PA12), metal (316L stainless steel), and polymer (thiol-ene) via numerous AM technologies, including stereolithography (SLA), multijet fusion (MJF), fused deposition modeling (FDM), direct metal laser sintering (DMLS), and selective laser melting (SLM). The developed deep-learning-driven genetic metaheuristic algorithm was able to achieve a particular strain value for a considered topology of the lattice structure by controlling the considered input parameters. For instance, in order to achieve a strain value of 2.8 × 10^−6^ mm/mm for the sea urchin structure, the developed model suggests the optimal stress (11.9 MPa), unit cell size (11.4 mm), total height (42.5 mm), breadth (8.7 mm), width (17.29 mm), and relative density (6.67%). Similarly, these parameters were controlled to optimize the strain for other investigated lattice structures. This framework can be helpful in designing various AM lattice structures of desired mechanical qualities.

## 1. Introduction

Lattice structures are a type of cellular material that has better thermomechanical properties than other cellular materials, such as foams. The improvement in AM technology has engrossed both academia and industry toward its deployment in real-world applications [1]. It is becoming easier and easier to design and construct cellular or lattice structures as metal additive printing technology resolution improves. Although there are few restrictions on the range of possible unit cell topologies, little is known about how the underlying unit cell topology influences the mechanical performance of lattice structures. A deeper comprehension of lattice structure performance based on unit cell topology can make it simpler to choose the appropriate unit cells to achieve the desired lattice structure mechanical properties [2].

In the natural environment, complex topological structures such as cellular structures, porous structures, and lattice structures are particularly prevalent [3]. The majority of these complex structures are nonzero genus geometries, which feature a lot of voids or holes. Complex topologies provide significant benefits for properties. The geometric model’s weight can be considerably lowered by using a lot of holes, and this can also minimize the amount of material, energy, and time needed to manufacture it. These optimized structures can be used to absorb energy, attenuate sound, isolate vibrations, or dissipate heat. Despite having many benefits, complex topological structures are challenging to manufacture using conventional production methods. Researchers have tried a number of methods (other than additive manufacturing) to create complicated topological structures [4]. Yet, these techniques fall short in terms of controlling the final pore characteristics. No matter what the shape geometry is, the most advanced method to fabricate complicated topological structures is AM [5]. A variety of materials can even be utilized to build complex topological structures thanks to advances in AM fabrication technology. Due to the ease with which complex lattice structures may be created using AM technology, both business and research now have the option of free-form production. Many material scientists have looked at the strength of lattice structures in relation to things such as the kind, size, and relative density of lattice cells, among other things. An extensive amount of research has been conducted to investigate the deformation behavior of different materials and structures [6,7,8]. Other than experiments, several models have been proposed to study the fatigue behavior of defective and notched components [9,10,11].

Nonetheless, the design processes for complicated topology structures face new difficulties as a result of AM technology. To design the most efficient and complex design for AM fabrication technology, the computer-aided design (CAD) technique has been used for a long time [12,13]. The two most often used techniques for representing 3D geometry are mesh models and parametric models. Nevertheless, not all complex topological structures can be designed using them. Several meshes have been employed for the approximation of a planned model, which is extremely resource-intensive, in order to produce correct modeling results. Additionally, merging the entire geometry would require dozens of parametric surfaces, which could also have an adverse effect on the outcome [14,15]. Regarding the design task, several design needs, such as mechanical qualities, printability, and other particular demands, should be taken into consideration. To cope with all these AM fabricated topologies of lattice structures, artificial intelligence and machine learning can be considered a compatible solution.

Artificial intelligence (AI) has opened up new avenues for improved design, process management, and quality assurance in additive manufacturing. Numerous engineering applications could benefit from the efficiency, effectiveness, and decision-making processes that artificial intelligence and machine learning techniques could potentially improve [16,17,18,19]. These techniques have proven their effectiveness for prediction [18,20,21], identification and monitoring [17,19], design optimization [22,23], control, sampling [24], data augmentation and characterization [25], and other tasks. However, the data scarcity in AM severely hinders the widespread application of AI approaches [26]. Few AI-based attempts have been made in recent years to predict the mechanical properties of AM lattice structures using AI. For example, recently, a deep neural network (DNN) model was developed and tested to evaluate the stress–strain association of the AM lattices [27]. The authors reported that their model was able to yield an accuracy of R^2^ = 0.936. They also performed a parametric study and interpreted the model’s predictions through explainable AI or XAI (explainable artificial intelligence) and highlighted the most sensitive features to estimate the strain of the AM lattices in their considered data range. In an earlier investigation, the same group of authors performed a short literature review and then proposed an artificial neural network (ANN) model with great accuracy (R^2^ = 0.99) to evaluate the mechanical behavior of AM lattices [28]. Their developed model’s predictions were true for several topologies on a lot of materials and created by various AM methods. In another study [29], to anticipate the inherent strain for powder bed fusion additive manufacturing based on a hatch pattern, neural networks were built. The findings demonstrated that the trained neural network could accurately anticipate the intrinsic strain of any given hatch pattern. Other than ANN and DNN, convolutional neural networks (CNNs) have been developed and evaluated with a respectable degree of accuracy for the prediction of geometric deviation in additive manufacturing [30].

The foregoing literature review suggests that no comprehensive framework has been developed to optimize the strain of numerous AM lattice structures (built on various materials and AM techniques) by controlling their design and process parameters. Therefore, in order to fill this research gap, the objective of this study was to propose a hybrid data-driven metaheuristic framework to optimize the strain of various topologies such as sea urchin (open cell) structure, honeycomb, and Kelvin structures simple, round, and crossbar (2 × 2) that were fabricated using different materials such as plastics (PLA, PA12), metal (316L stainless steel), and polymer (thiol-ene). The metaheuristic framework is driven by an optimal DNN model aided by a Bayesian surrogate model.

## 2. Materials and Methods

**Figure 1:**
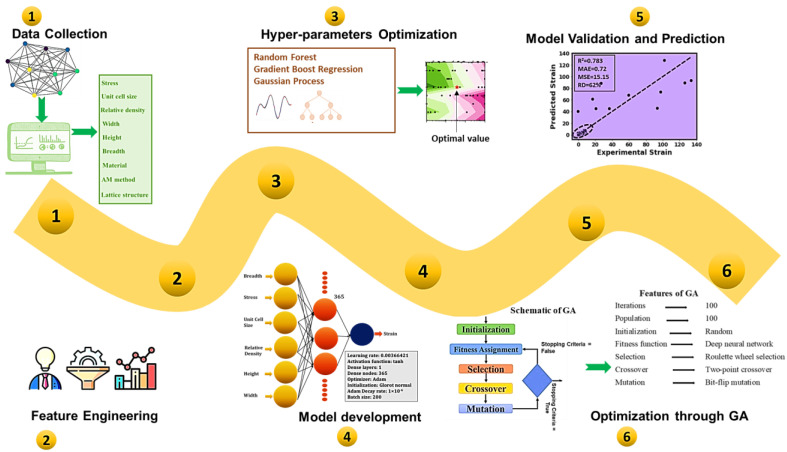
A flowchart of the proposed methodology for strain optimization of the AM lattice structures.

In this work, a hybrid framework using a deep-neural-network-based genetic algorithm (DNN-GA) was developed to find the values of the considered design and process parameters (including stress, total height, breadth, width, relative density, and unit cell size) of five additively built lattice topologies for a particular or optimal strain value. The considered topologies of lattice structures for the strain optimization included sea urchin (open cell) structure, honeycomb, and Kelvin structures simple, round, and crossbar (2 × 2). The overall methodology to optimize the strain of the considered AM lattice structures by controlling the impactful design and process parameters is depicted in Figure 1. Figure 1 contains six steps or phases. The first five steps comprised data collection, data preprocessing, feature engineering, hyperparameters tuning, development, and validation of the DNN model. In the last step, a hybrid DNN-GA framework was developed to optimize the investigated output.

The detailed procedure to develop the deep neural networks model (including data collection, data preprocessing, feature engineering, hyperparameters tuning, development, and validation of the DNN model) has already been provided in our prior study [27]. Herein, a short description is provided as a reference. In the first step, the data are collected for the considered AM lattice structures from the literature to develop the DNN model [27]. As can be seen, the collected data contains various input parameters (as listed in Figure 1) and the strain. In the second step, the collected data are cleaned, and then different data distribution methods (normal distribution, nth root transformation, and robust scaler) are tried. In this step, the collected data are also normalized using the Min–Max Scaler. In order to find the most important parameters as the inputs of the model, feature engineering and data correlation (Pearson correlation heat maps) are used. After that, different Bayesian surrogate models, including random forest (RF), gradient boost regression trees (GBRTs), and Gaussian process (GP), were used to find the optimal set of hyperparameters (such as activation function, learning rate, decay rate, optimizer, number of iterations, number of dense nodes and layers, etc.) to develop the accurate DNNs (deep neural networks) model. The optimal hyperparameters include a learning rate of 0.003664218944047891 and a single hidden layer with 365 dense nodes. The DNN model makes use of “tanh” as the activation function, “glorot normal” as the initialization mode, and “Adam” as the optimizer, while the decay rate and batch size are 1 × 10^−6^ and 200, respectively. The overall structure of the final DNN model includes an input layer of six neurons, a dense layer of 365 nodes, and an output layer of a single neuron [27]. Explainable artificial intelligence (XAI) via the SHAP library was employed to find the contribution of each input parameter to estimate the strain of the considered lattice structures. The results of the XAI are also important to verify the significance of the input parameters selected for the DNN model. The detailed methodology of training and developing an optimal DNN model can be found in our prior studies [27,31].

**Figure 2:**
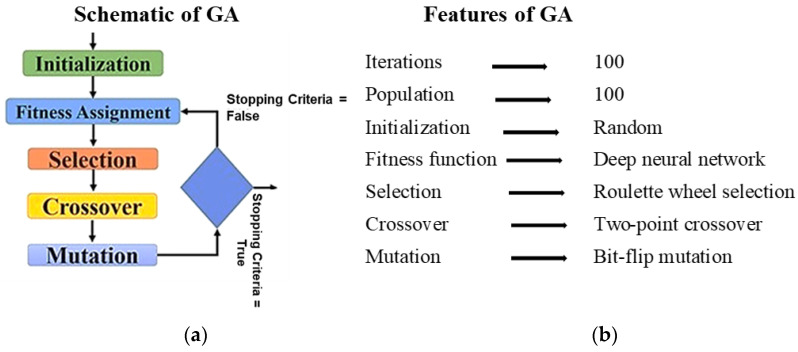
(**a**) Schematic and (**b**) features of developed metaheuristic framework.

The developed DNN model serves as a fitness function to build the metaheuristic (GA) model, which is finally used to yield the optimal strain by maximizing/minimizing the employed input parameters. The genetic algorithm is a stochastic search algorithm that draws its inspiration from the fundamental biological ideas of biological evolution, according to which only the strongest living things prevail over the weaker ones. It imitates evolutionary processes, such as mutation, crossover, and selection. Each member of the population, which initially comprises individuals of diverse fitness levels, is made up of a variety of traits, attributes, and genes. Later, each person’s fitness is assessed, and the fittest ones reproduce new offspring who inherit their ancestors’ expertise. Then, individuals with random features are used for mutation, and the founding parents are discarded. Until the ideal result is attained or the maximum number of iterations has been achieved, the procedure is repeated. A schematic of the GA algorithm is shown in Figure 2.

## 3. Results

This study considered five topologies of AM lattice structures, including sea urchin structure, honeycomb, and Kelvin structures simple, round, and crossbar (2 × 2). The results and discussion section explain the best stress, unit cell size, total height, breadth, width, and relative density values for a given value of strain for the considered topologies. A detailed discussion is made in the succeeding paragraphs of the results and discussion section.

### 3.1. Optimization of the Sea Urchin Structure

In our study, the considered sea lattice structures were fabricated by PLA material using fused deposition modeling (FDM). The input parameters were tuned in a minimum and maximum value, and the range is as mentioned in Table 1.

**Figure 3:**
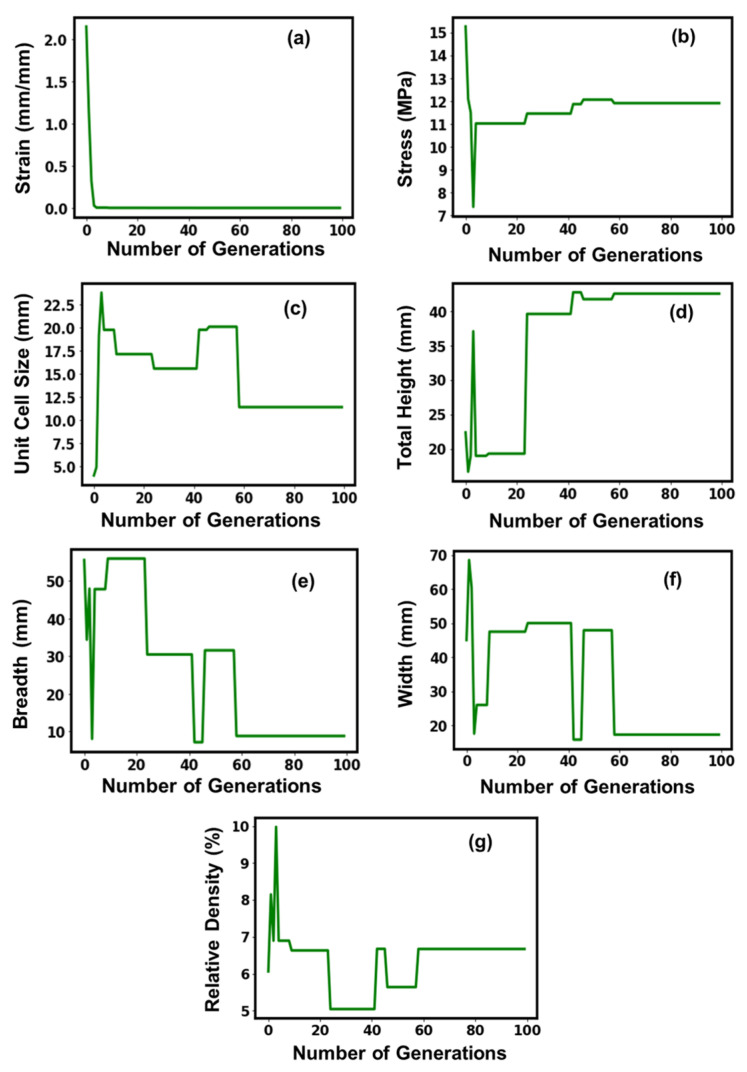
Suggested values of optimal parameters (**a**–**g**) for the sea urchin lattice structure.

Table 2 shows the attained optimal parameter values of the sea urchin structures, where an optimal strain value of 2.8 × 10^−6^ mm/mm was attained. The ideal total height and relative density determined for the sea urchin structures were noticeably larger than those for the other lattice structures under consideration, coming in at 42.5 mm and 6.67%, respectively. The sea urchin structure, on the other hand, had the smallest optimal breadth of any of its corresponding lattice structures at 8.7 mm. Figure 3 depicts the GA model’s convergence as the training process’s number of generations increased. The strain curve exhibited a smooth, exponentially degrading tendency before reaching a stable and ideal value, which was attained around the fifth executable generation at a strain value of about 2.8 × 10^−6^ mm/mm. The stress optimal value, which plateaued off after crossing the 60th executable generation and stabilized at 11.9 MPa, initially exhibited a sharp decline up until the progress of the 5th executable generation, after which it exhibited a marked improvement and reached its peak. Contrarily, the unit cell size fluctuation tendency was highly variable, reaching a peak of about 23 mm on the 5th executable generation before undergoing a stepwise decrease and a peak before displaying stable behavioral tendencies after the 60th executable generation. Although it begins to decrease sharply after the first few generations, the total height shows a noticeable increase after the 25th executable generation, following which it flattens out and stabilizes at 42.5 mm after the 60th executable generation. With regard to their fluctuating tendencies before experiencing a second crest around the 40th executable generations, where it flattened out and displayed a steep decline before stabilizing off at a value of the 60th executable generations, the behavioral tendencies of breadth, width, and relative densities with the number of executable generations were more or less similar. Overall, there were variations in the values for all parameters, which is understandable given that the model is still learning, but all the parameters stabilized throughout the course of the 60th executable generation.

### 3.2. Optimization of the Honeycomb Structure

Honeycombs are two-dimensional periodic cellular materials that are relatively strong and stiff along the microstructural normal but flexible and weak in the plane. This material is ideal for weight-bearing applications, especially in the automotive and aerospace industries, in which components with a high strength-to-weight ratio are required. However, designing honeycomb structures using conventional machining processes is a difficult and time-consuming task. In this respect, it would be interesting to study the capabilities of the AM process, as AM appears to be an alternative solution due to its design freedom [32]. Herein, the investigated honeycomb structures were fabricated using PA12 material using multijet fusion (MJF) fabrication technology. Table 3 shows the value of the optimal parameters obtained for the honeycomb structures. It can be seen that for the honeycomb structure, the optimal value of the strain was around 6.5 × 10^−6^ mm/mm, and it was also characterized by the highest optimal value of width for the studied lattice structures corresponding to 69.89 mm and by the least unit size corresponding to 1.5 mm. The results obtained can be said to be in accordance with the findings of [33], as the smaller unit sizes in the honeycomb structures exhibited better stiffness and, thus, exhibited a much more stronger response under compression.

**Figure 4:**
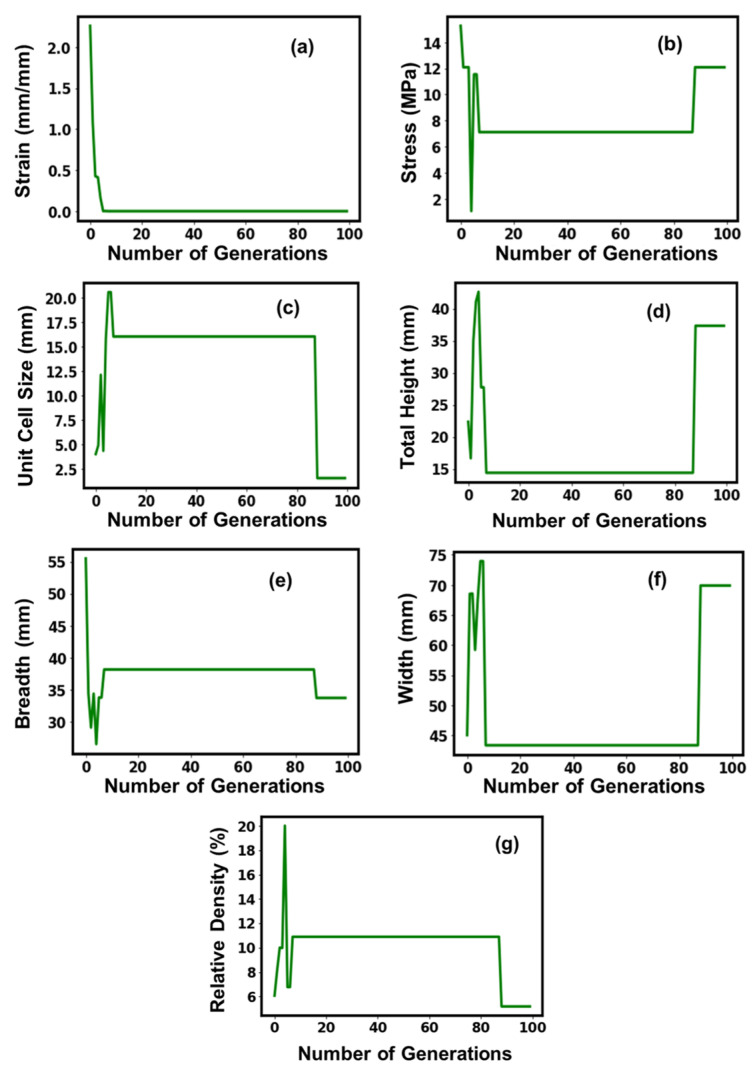
Suggested values of optimal parameters (**a**–**g**) for the honeycomb lattice structure.

Figure 4 shows the convergence of the GA model for the honeycomb structures, with an increase in the number of generations during the training process. Initially, the model showed fluctuations in the values for all parameters during the learning phase but showed stabilizing tendencies afterward with the execution of the generations. The strain curve showed a similar exponential decay with the execution of the generations and stabilized around an optimal value of 6.5 × 10^−6^ mm/mm, which was executed around the 5th generation and showed a stabilizing behavioral tendency afterward. The dependency of the stress and the unit cell size with the number of executable generations exhibited similar behavioral traits, reaching a crest around the 10th executable generation, after which it showed a steep decline and flattened off, indicating a stable behavioral tendency, but around the 80th generation, the curves showed a steep increment and decrement, respectively, before stabilizing and providing an optimal value of 12 MPa and 1.5 mm, respectively. The remaining parameters, namely, total height, breadth, width, and relative density, exhibited plateau-like behavior until the 80th iteration and might give the impression of stabilizing behavior and attaining an optimal value. However, post the 80th executable generation, the total height, width, and relative density increased approximately 1.5–2 fold and flattened off, attaining an optimal value of 37.3 mm, 69.89 mm, and 5.18%, respectively, while the breadth experienced a significant decline and flattened off, attaining an optimal value of 33.69 mm.

### 3.3. Optimization of the Kelvin Simple 2 × 2 Structure

Due to their better mechanical qualities and simplicity of modeling, Kelvin structures have been the subject of much investigation [34]. A group of authors [35] investigated how Kelvin cells were affected by relative density and loading circumstances. For our case study, we investigated the simple Kelvin 2 × 2 structure by 316L stainless steel using selective laser melting (SLM) technology. Table 4 shows the value of the optimal parameters obtained for the Kelvin simple 2 × 2 structure. It can be seen that the simple Kevin 2 × 2 structure was characterized by the highest optimal strain and least optimal stress value compared with its lattice structure counterparts, which were 1.7 × 10^−5^ mm/mm and 4.15 MPa, respectively. The optimal value of the height parameter of the simple Kelvin 2 × 2 structure was also toward the lower optimum range, standing at a value of 28.5 mm. Amongst all the other compared lattice structures, this structure particularly showed more deformation behavior, having a high value of relative density that signified the deformation to be stretch-dominated, as per findings in the literature [36].

**Figure 5:**
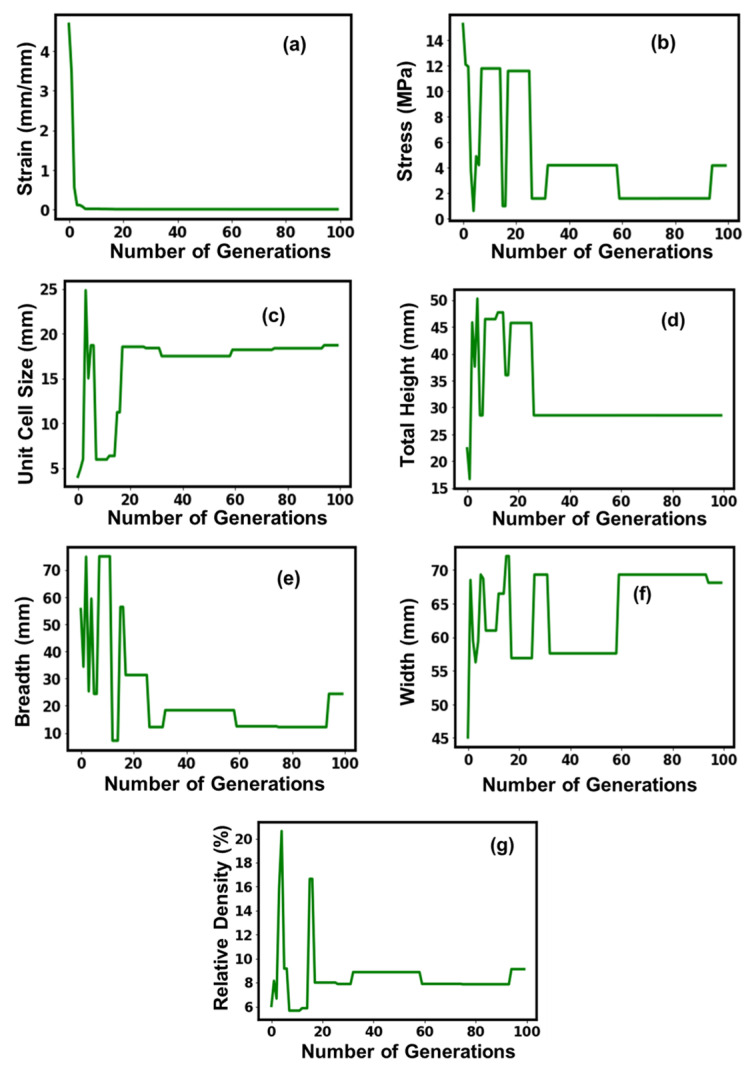
Suggested values of optimal parameters (**a**–**g**) for the Kelvin simple 2 × 2 structure.

Figure 5 shows the convergence of the GA model for the Kelvin simple 2 × 2 structure, with an increase in the number of generations during the training process. Although initially, all the parameters displayed a tendency to fluctuate, after the 60th executable generation, a more or less consistent behavior was established in all situations. The stress curve displayed initial fluctuations that formed plateau-like crests, with the executable generations showing a significant decline in crest amplitude until the 60th executable generation, before stabilizing until the 80th executable generation and before showing a significant increment by two folds and flattening.

The variation in the width and relative density with the number of executable generations showed a similar tendency in terms of having initial fluctuations in the learning phase, showing stabilizing tendencies in the 30th–60th executable generations before an increment by 1.2-fold and coming to stabilized conditions, signifying attainment of the optimal value. The total height and unit cell size achieved stable behavior with the execution of the 20th generation, while the breadth value, although it showed mostly detrimental fluctuations, stabilized to an optimum value of 24.32 mm post the 80th executable generation.

### 3.4. Optimization of the Kelvin Round 2 × 2 Structure

As pointed out in the literary work of [34], the formation of Kelvin round 2 × 2 structures from simple Kelvin 2 × 2 structures can be attributed to the addition of fillets. A group of researchers [35] studied the impact of loading circumstances and relative density on Kelvin cells. They observed that the strength of the structure grew as the relative density of the structure increased. Additionally, the cells’ method of deformation switched from cell edge bending to cell membrane bending. In our study, the considered structure was made with the material Visi Jet SL Clear, and the fabrication methodology used was the SLA technique. Table 5 shows the value of the optimal parameters obtained for the Kelvin round 2 × 2 structure.

**Figure 6:**
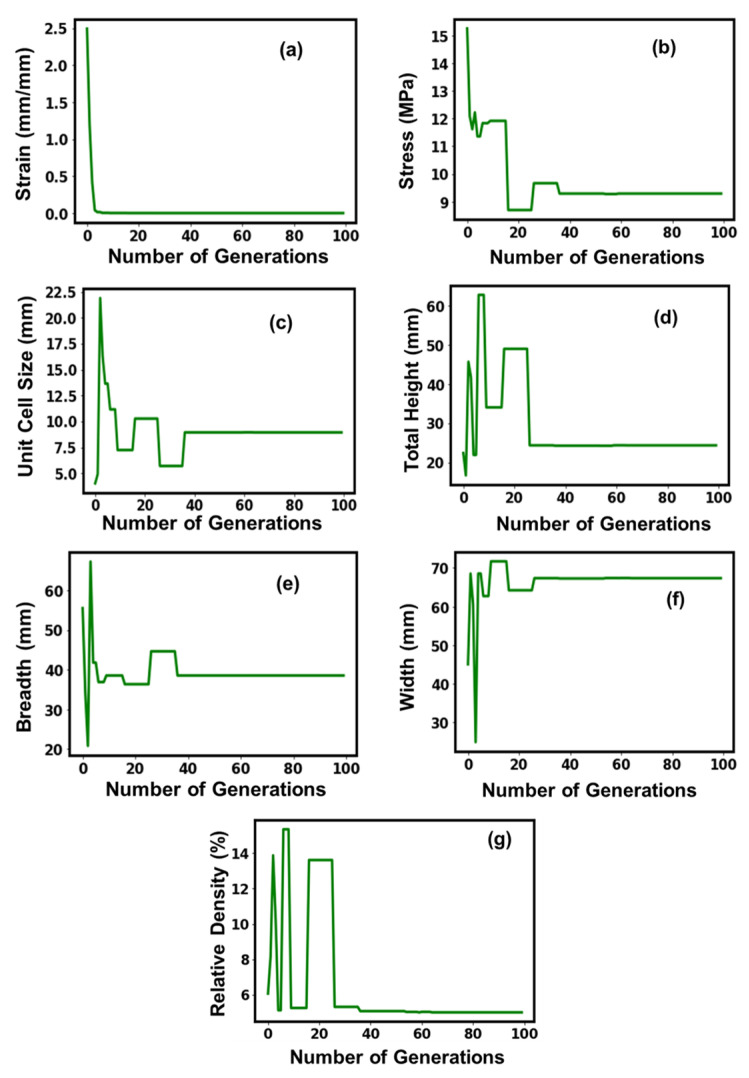
Suggested values of optimal parameters (**a**–**g**) for the Kelvin round 2 × 2 structure.

It is evident that the stress value increased almost two-fold when compared with the straightforward 2 × 2 Kelvin structure, whereas the relative density and unit size both experienced a sharp decline by 1.08 times and 0.22 times, respectively. Thus, it is clear that the additional fillets in this situation improved the lattice structure’s rigidity and resilience, which was already reported [37]. Moreover, the smaller optimal unit size ensured better stiffness exhibition, as pointed out by [38]. Figure 6 shows the convergence of the GA model for the Kelvin round 2 × 2 structure. While the initial fluctuations in the learning phase of the model are noted here as well, overall, the model exhibited a stable behavior after the 40th executable generation, which is similar to the previous characteristic curves for the previously stated lattice structures. When it came to the breadth and relative density, there was a similar propensity for them to fluctuate up until the 40th executable generation, at which point there was a plateau peak that lasted about from the 10th to the 20th executable generation. The strain curve exhibited an exponential decline relationship with the passage of the executable generations that was more or less identical. Up to the 40th executable generation, when it stabilized, the stress value exhibited a more or less significant decline from the initialized executable generations. Comparatively quicker than the other lattice structure characteristics, the parameter of total height obtained a somewhat boosted optimization at the 25th executable generation.

### 3.5. Optimization of Kelvin Cross Bar 2 × 2 Structure

The Kelvin-type lattice structure has been widely investigated owing to its mechanical qualities and ease of modeling [39]. In our study, the investigated Kelvin cross bar 2 × 2 structure was manufactured with the help of a thiol-ene polymer using the additive manufacturing methodology of direct metal laser sintering (DMLS). Table 6 shows the value of the optimal parameters obtained for the Kelvin cross bar 2 × 2 structure.

**Figure 7:**
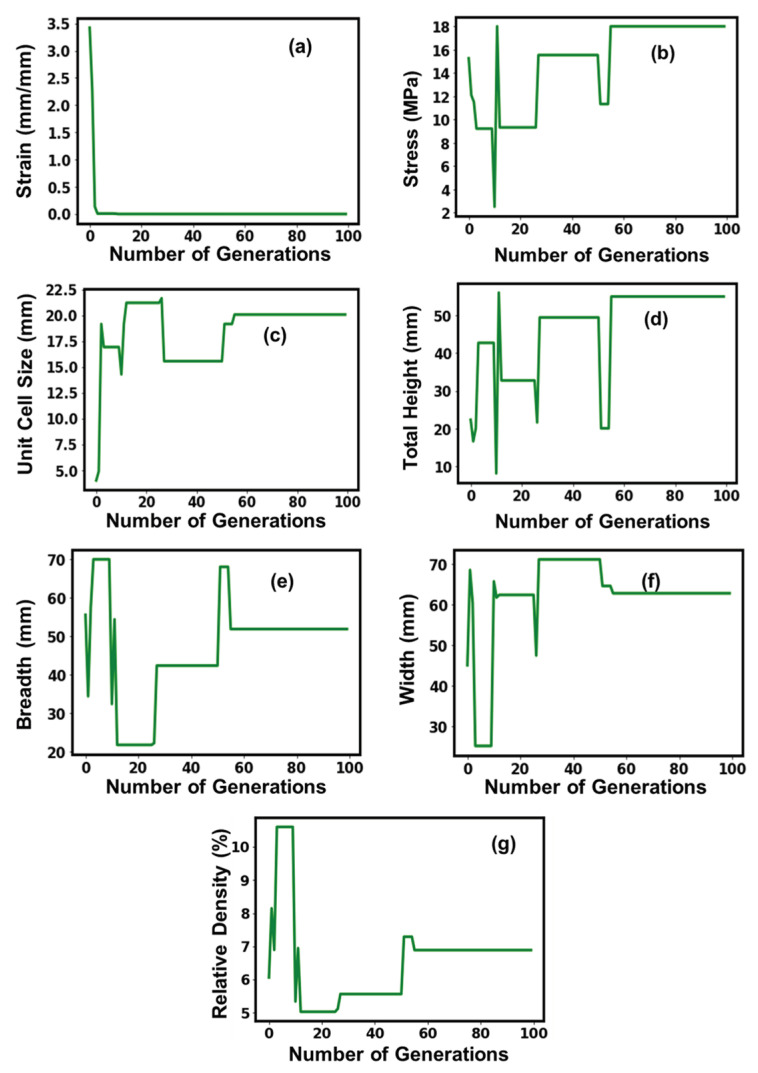
Suggested values of optimal parameters (**a**–**g**) for the Kelvin cross bar 2 × 2 lattice structure.

The optimal parameters achieved for the Kelvin cross bar 2 × 2 structure exhibited a higher value for optimal stress as well as a lower value for optimal strain, demonstrating the fact that the structure is more resistant to deformation of any kind. The structure is also distinguished by the largest relative density among the equivalents taken into consideration, suggesting that, contrary to what is suggested in literary works, the lattice structure is primarily a stretch structure [36]. Given that the unit cell size was significantly bigger in this case and was the largest among the counterparts of the investigated lattice structures, it is thought that reducing the unit cell size may be a strategy that can be revisited in order to further improve the mechanical properties of the structures. The geometry of the proposed lattice structure was also larger than that of the prior structure, indicating that it was far more spread out than its predecessors. Figure 7 shows the convergence of the GA model for the Kelvin cross bar 2 × 2 structure. On an overall basis, it can be seen that the value fluctuations at the learning phase of the model continued until the execution of the 60th executable generation, after which the model gradually stabilized. The fluctuations and stabilization characteristics for the relative density and width with the executable generations were almost identical to one another, with both stabilizing at around the 60th executable generation. The variation in stress, unit cell size, and total height with the executable generation initially showed a fluctuating behavior until the 20th executable generation, after which it displayed plateau-like secondary stabilizing behavior followed by a significant increment or decrement to achieve the final stabilizing behavior at the proximity of the 60th executable generation. The breadth characteristics, in this case, exhibited a more volatile fluctuation behavior before achieving a stabilized value.

## 4. Conclusions

In this study, numerous topologies of AM lattices, including sea urchin (open cell) structure, honeycomb, and Kelvin structures simple, round, and crossbar (2 × 2), were considered, which were fabricated using different materials such as plastics (PLA, PA12), metal (316L stainless steel), and polymer (thiol-ene) via numerous AM technologies, including FDM, MJF, SLM, SLA, and DMLS. A deep-learning-driven genetic metaheuristic model was developed to find the best values of the considered process and design variables for a given value of strain for a certain topology of the lattice structure. This study’s key findings are as follows.

− For the sea urchin structure, the developed model suggests the optimal stress (11.9 MPa), unit cell size (11.4 mm), total height (42.5 mm), breadth (8.7 mm), width (17.29 mm), and relative density (6.67%) for a strain value of 2.8 × 10^−6^ mm/mm.
− For the honeycomb structure, the proposed model finds the optimal stress (12 MPa), unit cell size (1.5 mm), total height (37.3 mm), breadth (33.69 mm), width (69.89 mm), and relative density (5.18%) for a strain value of 6.5 × 10^−6^ mm/mm.
− For the Kelvin simple (2 × 2) structure, the developed model suggests the optimal stress (4.15 MPa), unit cell size (18.6 mm), total height (28.5 mm), breadth (24.32 mm), width (68.12 mm), and relative density (6.12%) for a strain value of 1.7 × 10^−5^ mm/mm.
− For the Kelvin round (2 × 2) structure, the proposed model finds the optimal stress (9.2 MPa), unit cell size (8.9 mm), total height (24.3 mm), breadth (38.5 mm), width (67.32 mm), and relative density (5.007%) for a strain value of 6.1 × 10^−6^ mm/mm.
− For the Kelvin cross bar (2 × 2) structure, the developed model suggests the optimal stress (17.99 MPa), unit cell size (20.06 mm), total height (54.8 mm), breadth (51.7 mm), width (59.7 mm), and relative density (9.89%) for a strain value of 6.2 × 10^−6^ mm/mm.

These findings can be used to develop AM lattice structures by controlling the design and process parameters for other topologies and manufacturing methods on different materials by including the relevant, impactful, deep-learning-driven genetic metaheuristic algorithm.

## Figures and Tables

**Table 1:** Range of the variables.

Input Parameters	Minimum	Maximum
Stress (MPa)	0	342.32
Unit cell size (mm)	1.5	25
Total height (mm)	7	75
Breadth (mm)	7	75
Width (mm)	7	75
Relative density (%)	5	90

**Table 2:** Optimal parameters for sea urchin structure.

Parameter	Value
Stress (MPa)	11.9
Unit cell size (mm)	11.4
Total height (mm)	42.5
Breadth (mm)	8.7
Width (mm)	17.29
Relative density (%)	6.67
Strain (mm/mm)	2.8 × 10^−6^

**Table 3:** Optimal parameters for honeycomb structure.

Parameter	Value
Stress (MPa)	12
Unit cell size (mm)	1.5
Total height (mm)	37.3
Breadth (mm)	33.69
Width (mm)	69.89
Relative density (%)	5.18
Strain (mm/mm)	6.5 × 10^−6^

**Table 4:** Optimal parameters for Kelvin simple (2 × 2) structure.

Parameter	Value
Stress (MPa)	4.15
Unit cell size (mm)	18.6
Total height (mm)	28.5
Breadth (mm)	24.32
Width (mm)	68.12
Relative density (%)	6.12
Strain (mm/mm)	1.7 × 10^−5^

**Table 5:** Optimal parameters for Kelvin round (2 × 2) structure.

Parameter	Value
Stress (MPa)	9.2
Unit cell size (mm)	8.9
Total height (mm)	24.3
Breadth (mm)	38.5
Width (mm)	67.32
Relative density (%)	5.007
Strain (mm/mm)	6.1 × 10^−6^

**Table 6:** Optimal parameters for Kelvin cross bar (2 × 2) structure.

Parameter	Value
Stress (MPa)	17.99
Unit cell size (mm)	20.06
Total height (mm)	54.8
Breadth (mm)	51.7
Width (mm)	62.7
Relative density (%)	9.89
Strain (mm/mm)	6.2 × 10^−6^

## Data Availability

The data are contained within the article.
